# Understanding differences in HIV/HCV prevalence according to differentiated risk behaviors in a sample of PWID in rural Puerto Rico

**DOI:** 10.1186/s12954-016-0099-9

**Published:** 2016-03-08

**Authors:** Roberto Abadie, Melissa Welch-Lazoritz, Camila Gelpi-Acosta, Juan Carlos Reyes, Kirk Dombrowski

**Affiliations:** Department of Sociology, University of Nebraska-Lincoln, 206 Benton Hall, Lincoln, NE 68588 USA; Social Science Department, LaGuardia Community College, 31-10 Thomson Ave., Long Island City, NY 11101 USA; Department of Biostatistics and Epidemiology, University of Puerto Rico, 365067, San Juan, PR 00936 USA

**Keywords:** HIV-HCV risk factors, PWID, Sharing, Injection equipment, Rural Puerto Rico

## Abstract

**Background:**

Blood contained in needles and injection equipment has been identified as a vector for HIV and HCV transmission among people who inject drugs (PWID). Yet, there is often a wide discrepancy in prevalence for both viruses. While microbiological differences between viruses influence prevalence, other variables associated with the way drugs are acquired and used, also play a role.

**Methods:**

Respondent-driven sampling (RDS) methods recruited a sample of 315 current intravenous drug users in rural Puerto Rico. Information about type and frequency of use, HIV and HVC risk behaviors (sharing needles, cookers, cotton, and water), sexual behaviors, and alcohol use was collected. HIV and HCV statuses were assessed via rapid antibody tests. *T* tests compare means of participants who tested positive (reactive) to those who tested negative. Logistic regression analyses were used to validate the association of the risk factors involved.

**Results:**

Tests showed a significant difference in HIV (6 %) and HCV (78.4 %) prevalence among a population of current PWID. The main risk behaviors in HCV transmission are the sharing of injection “works”, (e.g., cookers, cotton, and water). Sharing works occurred more than twice as often as the sharing of needles, and HCV+ and HCV− individuals reported the same needle sharing habits.

**Conclusions:**

Washing and rinsing injection works with water seems to prevent HIV transmission, but it is unable to prevent HCV infection. While education about the need to clean injection equipment with bleach might be beneficial, equipment sharing—and the subsequent risk of HVC—might be unavoidable in a context where participants are forced to pool resources to acquire and use intravenous drugs.

**Electronic supplementary material:**

The online version of this article (doi:10.1186/s12954-016-0099-9) contains supplementary material, which is available to authorized users.

## Background

The current 2015 study of people who inject drugs (PWID) in rural Puerto Rico found that 6.0 % of the sample was HIV positive while 78.4 % of the sample was Hepatitis C (HCV) positive using rapid tests. Research has shown that PWID in Puerto Rico are at a very high risk for HIV, as injection drug use is the most important factor behind the prevalence of HIV on the island [1–4]. Puerto Rico occupies the sixth place in HIV prevalence among all US states and territories [5]. Injection drug use is responsible for almost half of the accumulated AIDS cases and nearly 26 % of HIV cases diagnosed between 2005 and 2011. In 2010, PWID accounted for 8.6 % of new HIV infections across the USA, while the proportion in Puerto Rico was over two times greater (20.4 %) [6]. A study by Reyes et al. among PWID in the metropolitan San Juan area places HIV prevalence at 17 % and HCV prevalence at a striking 89 % [7].

Studies have documented wide discrepancies in the prevalence of HIV and HCV among PWID across the world [8, 9]. Research has identified the main causes of transmission for both viruses as blood contained in the syringes, cookers, cotton, and water [10, 11]. Still, PWID appear to be more vulnerable to HCV than to HIV. The microbiological properties of HCV are considered one of the main reasons behind these prevalence discrepancies [12]. Indeed, HCV is more potent and resilient than the HIV virus and can live on surfaces of the body for up to 6 weeks while maintaining infectivity [13, 14]. Years of injection, using in shooting galleries, incarceration, number of injection partners, and history of sexually transmitted diseases have been associated with higher rates of HCV acquisition among PWID [15–17]. In Puerto Rico, lack of knowledge of the HCV and lack of awareness of HCV serostatus have also been identified as factors behind transmission [18].

Various researchers [19, 20] have suggested that the sharing of injection equipment, such as cookers and cotton, could be contributing to HCV transmission vulnerability for PWID. Indeed, many argue that PWID may be sharing cookers, cottons, and water more often than they are sharing syringes, and this is particularly true since the advent of needle exchange programs in the USA [21]. Another study with PWID in Denver, CO, found that while just 22 % of users shared needles, 86 % used a common cooker to divide drugs [22].

Our study seeks to explore these risk factors among rural Puerto Rican PWID, a population whose risk behaviors have never before been studied, perhaps due to the difficulties of accessing a marginalized population. We also aim to contribute to the literature by comparing the risk behaviors of HCV positive and HCV negative PWID in rural Puerto Rico. By outlining the similarities and differences in risk behaviors between these two groups, we will help identify the service needs to be addressed to arrest the further spread of HCV among PWID in Puerto Rico and abroad.

## Methods

### Data

This paper utilizes data from 315 PWID residing in Cidra, Comerio, Aguas Buenas, and Cayey, four rural towns in the mountainous area of central Puerto Rico, about 30–40 miles from San Juan. Sites were selected because they were representative of rural PWID on the island [23]. In addition, these sites were chosen due to the presence of El Punto en la Montaña, the only syringe exchange program operating in rural Puerto Rico, with whom we established a close collaboration that facilitated data collection with this population. Interviews were completed between April 2015 and June 2015. Sample recruitment was managed using respondent-driven sampling (RDS) and we started two seeds in each of the four towns (for a total of 8 seeds, 307 recruits). Participants who completed the survey were given three referral coupons to pass out to other PWID they knew and who had not previously participated in the study. Every eligible referral earned the recruiter an additional $10. Upon completion of the questionnaire, participants were given $25. RDS has proven effective in recruiting hard to reach populations [24–27]. Participants were 18 years of age or older, alert at the time of the interview, and had injected drugs within the past 30 days. Verification of current injection use was done through visual inspection of injection track marks as well as through a questionnaire that measuring knowledge of injection practices [28, 29].

The questionnaire was interviewer-administered and based off of the CDC NHBS IDU Round 3 Questionnaire version 13. In addition to demographic variables, we collected information about type and frequency of drug use, as well as HIV and HCV risk behaviors such as sharing of needles, cookers, cotton, and water in addition to sexual behaviors and alcohol use. HIV and HCV status was assessed through the use of INSTI Rapid HIV antibody tests (Biolytical Laboratories) and OraQuick HCV Rapid antibody tests (OraSure Technologies). Every participant was compensated an additional $5 for each rapid test performed. Participants who tested positive for HCV or HIV were offered referral and transportation to a primary care doctor for confirmatory testing. The study received IRB approval through the University of Nebraska-Lincoln (IRB# 20131113844FB) and the University of Puerto Rico School of Medicine (IRB# A8480115).

### Measures

HIV status and HCV status were determined by the results of the INSTI Rapid HIV and OraQuick Rapid HCV test. *Annual per capita income* was assessed by two questionnaire items, one which participants selected an income bracket and a second which participants reported how many people rely on that income during the year. The upper limit of the income bracket was then divided by the number of people relying on income to achieve an approximation of annual per capita income. *Percent unemployed* was assessed using a question that asked participants which best described their employment status: employed full time, employed part time, full time student, retired, unable to work for health reasons, unemployed, or other.

*Frequency of injection* was assessed using the question “in the last 12 months, on average, how often did you inject drugs?” with response choices of (1) one time per month, (2) 2–3 times per month, (3) one time per week, (4) 2–6 times per week, (5) one time per day, (6) 2–3 times per day, and (7) 4 or more times per day. *Number of people used needle* is a continuous measure based on the question “with how many people did you use a needle after they injected with it?”, this question was framed in the context of both “in the past 12 months” and “in the past month”. *Number of people used works* is also a continuous measure from the question “with how many people did you use the same cooker, cotton, or water that they had already used?” with responses for past year and past month. *Number of people backloaded* is a continuous measure assessing with how many people the participant used drugs that had been divided with a syringe that they had already used (i.e., backloading, frontloading), in the past year and in the past month.

*Frequency of* (1) *used needle utilization*, (2) *used cooker utilization*, (3) *used cotton utilization*, *and* (4) *used water utilization* are four categorical measures asking how often, in the past year, the participant used (1) needles that someone else had already injected with, (2) a cooker that someone else had already used, (3) a cotton that someone else had already used, and (4) water that someone else had already used. The response options are never (coded as 0), rarely (coded as 1), about half of the time (coded as 2), most of the time (coded as 3), and always (coded as 4).

### Analytic approach

Analysis of the RDS data was undertaken using both of the current, accepted RDS analysis platforms: RDSAT version 7.1 [30] and RDS Analyst [31]. No significant differences in results were found between the two routines. Dual homophily scores were calculated for the main demographic variables, including gender, age, HIV and HCV status, drug treatment participation, drug choice, number of sex partners, geographic location of recruitment, frequency of drug use, income, and homelessness in the past year. For the demographic variables (age, gender, income, location, and homeless status), no significant biases were discovered. Complex homophily results were found for individual variable values on drug of choice (only use of speedball was significant, other drugs were not), HCV status (only known positive status was significant) and treatment participation. As none of these influenced the demographic variables, the analyses discussed here were made from sample point estimates, unadjusted by RDS results*.* A full discussion of the RDS sample and its analysis is currently under review in the Puerto Rican Public Health Sciences Journal.

The results reported in this analysis stem from *t* tests to compare the means of participants who tested positive (reactive) for HCV antibodies in the rapid test, and participants who tested negative for HCV antibodies on several variables, including injection habits. Multivariate analyses using logistic regression were used to further evaluate the association of risk factors shown in the comparison of means tests. We conducted a logistic regression with each variable tested in Table 2 and controlled for gender (male), age, per capita income, education level (high school graduate or better), marital status (married or cohabiting), and number of years spent injecting drugs. All analyses were conducted using IBM SPSS Statistics software.

## Results

Table 1 presents demographic data for study participants. More than three quarters (78.4 %) tested positive for HCV during the rapid test, while 6.0 % tested positive for HIV. All 19 HIV positive participants were HCV co-infected. Approximately 90.5 % (*n* = 285) were male and 93.0 % were born in Puerto Rico. Almost all PWID who were not born in Puerto Rico had been born in the continental United States. The sample had a mean age of 41.8 years (range 18–70 years) and an average annual per capita income of $4452. Participants were mostly (85.4 %) unemployed, 21.9 % were homeless at the time of their interview, and 52.4 % were high school graduates (or higher). Only 2.9 % of participants were currently married, though an additional 19.3 % were living together as married, while 47 % were single and never married (the remaining 30.8 % were separated, divorced, or widowed).

**Table 1:** Descriptive statistics

	Mean/%	SD	*N*
% HIV positive (INSTI Rapid Test)	6.0 %		315
% HCV positive (OraQuick Rapid Test)	78.4 %		315
% Male	90.5 %		315
% Born in Puerto Rico	93.0 %		315
Age (years)	41.8	(10.07)	315
Annual per capita income	$4452	($3200)	311
% Unemployed	85.4 %		314
% Currently homeless	21.9 %		314
% Graduate high school (or higher)	52.4 %		315
% Married or living together as married	22.2 %		315
% Injected 4 or more times per day	39.7 %		315
% Injected 2–3 times per day	39.7 %		315

As shown in Table 2, commonalities in injection behaviors regardless of HCV status are apparent. For instance, both groups reported injecting drugs at the same average frequency during the past year. In addition, past year utilization of used needles and the number of people who used needles before them are exactly the same. However, significant differences between HCV statuses shed some light over the vast disparity between HIV and HCV infection found in rural Puerto Rico. First, HCV+ injectors are, on average, 3 years older than their HCV− counterparts (*p* ≤ .031). Second, those who were HCV+ began injecting drugs at a much younger age (20.7 years) than those HCV− participants (26.3 years; *p* ≤ .001); and third, HCV+ participants have spent 9.4 more years injecting drugs than their HCV− counterparts (*p* ≤ .001).

**Table 2:** Comparison of means for injecting practices by HCV status

	HCV+ (*n* = 247)	HCV− (*n* = 68)	*N*
Age (years)	41.8*	38.8*	315
Age at first use of injection drugs	20.7***	26.3***	315
# of years injecting drugs	21.9***	12.5***	315
Past year:			
Frequency of injection	5.9	5.9	315
# of people used needle	1.2	1.2	314
# of people used works	4.9	3.0	314
# of people backloaded	1.5	0.91	311
Frequency of used needle utilization	0.4	0.4	315
Frequency of used cooker utilization	1.1*	0.8*	315
Frequency of used cotton utilization	0.8	0.6	315
Frequency of used water utilization	0.7	0.5	315
Past month:			
# of people used needle	0.4	0.5	315
# of people used work	2.0**	1.0**	315
# of people backloaded	0.6	0.5	313

*p < .05, **p < .01, ***p < .001

Importantly, marked differences in their use of works are of note. Specifically, HCV+ participants used injection preparation equipment (cooker, cotton, and/or water) after an average of almost 2 more people in the past year than their HCV− counterparts (4.9 people compared to 3.0 people, *p* ≤ .122; though not significant at standard *p* values, this large difference is notable). In the past year, HCV+ participants backloaded after an average of .6 more people (*p* ≤ .086) and injected with a used cooker significantly more frequently (*p* ≤ .034) than HCV− participants. In the past month, HCV+ injectors used works after 1 more person than those without HCV (*p* ≤ .001).

After controlling for gender, age, annual per capita income, marital status, and level of education, the significance of the results from the comparison of means holds up (the entire logistic regression table is available online as Additional file 1 to this paper). Logistic regression results indicate that for every one-unit increase in the frequency of past year used cooker utilization, there is a 26 % increase in the odds of being HCV+ (*p* ≤ .029). Each additional person who used works before the participant during the past month is associated with an 18 % increase in the odds of being HCV+ (*p* ≤ .039). Interestingly, no measurements of needle sharing behaviors were statistically significant predictors of HCV.

The number of people who used works before a participant in the last year (4.9 HCV+ and 3.0 HCV−) is more than double (or quadruple for HCV+ injectors) the number of people who used a needle before a participant in the past year (1.2 for both HCV+ and HCV−). Similarly, the past year frequency of injecting with a used cooker was at least double that of injection with a used needle. Clearly, needle sharing was not the main mechanism for HCV transmission among rural Puerto Rican PWID.

## Discussion

This study found an HIV prevalence of 6 % and an HCV prevalence of 78.4 % among a sample of intravenous drug users in rural Puerto Rico. This prevalence for HCV is among the highest in the world [32–35]. A systematic review of 77 countries found a mid-point prevalence estimate for HCV among PWID between 60 and 80 % in 25 countries and more than 80 % in 12 countries [36].

Our results indicate that HCV risk lies in sharing works, or specifically cookers, and this could be especially problematic for disease contraction because of HCV’s ability to live outside the body for many days [37], and its ability to spread with only a very small concentration of blood that could be found in drug using equipment, even after rinsing with clean water [38]. Age, the number of people who had used works before the participant, and frequency of used cooker utilization are all significantly higher for individuals with HCV. While sharing needles, cookers, cotton, and water has been outlined as a main risk for both HIV and HCV, our study shows that the main risk for HCV infection, given current behaviors, is associated with sharing cookers more than with using needles after somebody else had used them. This builds upon research [39–42] showing that an important proportion of HCV infections are likely attributable to cooker or cotton sharing.

Sharing works could very well be related to the rural injectors’ needs to pool resources to acquire the drugs. The most commonly injected drug for our sample is speedball, a mix of heroin and cocaine, and most participants inject multiple times per day. In the four municipalities where study participants reside, the approximate cost of a small bag of heroin is $6, and $5 for a small bag of cocaine, thus requiring $11 for one single injection event. One means to deal with this is to pool resources and share the drugs. Further, speedball use is often associated with high frequency of injection [43]. This represents a significant challenge for the current sample of rural Puerto Rican injectors, who have an average per capita annual income of just $4452. When sharing, users mix the purchased heroin and cocaine bags in the same cooker before injecting. In fact, this practice is commonly called “caballo”, or horse, in rural Puerto Rico. Once the drug is dissolved in the cooker, injectors can collect their “share” via separate or shared syringe, taking turns. Others might prefer to backload. Dissolving the pooled drug in the same cooker may also be leading participants to using the same cotton to filter the drug.

In addition to the hardy virological properties of HCV, another factor contributes to the epidemic is lack of HCV treatment. While 82.5 % of our sample has state sponsored health insurance coverage (Reforma), many are unable to access HCV treatment, as the state health insurance plan will not cover this expensive treatment unless the patient is also HIV positive. Barriers to HCV treatment in the island have been also previously described [44] and as such, the vast majority of HCV patients carry the virus for many years [45].

Finally, interventions designed to provide more education to PWID about the particular viral characteristics of HCV and its strong transmission risk would be beneficial. Other strategies to reduce the prevalence of HCV among this population include more extensive testing and counseling, along with efforts to scale up HCV treatment for those that have tested positive to HCV, especially in resource-rich countries [46, 47]. However, while these interventions, as well as other more general harm reduction strategies like providing safe injection rooms [48] or extending the presence of needle exchange programs [49, 50] are advisable, the financial incentive to pool resources to jointly acquire and use the drugs continues to be a significant obstacle to curb the spread of HCV in this population. Furthermore, migration patterns, especially across borders, might complicate prevention efforts by making PWID harder to reach, or by introducing the risk of HIV/HCV infection in other social networks [51, 52].

### Limitations

One limitation resides in the fact that while RDS is a standard methodology for reaching marginalized populations, we cannot be sure that our sample completely accounts for the entire population of rural injectors. Another constrain is that this study asked participants about frequency of drug use at different intervals, spanning from the past month, to the past year. It is not possible to exclude the possibility of recall bias, especially when participants are asked to remember information from practices far removed from the present. Despite this limitation, we believe that probing participant about drug frequency at different time intervals is justified by the need to document potential changes in drug use patterns over time. This study is further limited in its reliance on self-reports rather than observed risk behaviors. While asking participants about their risk practices illuminates the risks they face in the course of injecting drugs, it remains a less precise alternative when compared with directly observing these same behaviors. The interpretation of data about co-infection of HIV and HCV is limited because our survey did not collect temporal data about which infection came first, obscuring the fact that PWID living with HIV (*n* = 19) are more likely to contract HCV.

## Conclusions

The wide discrepancy in prevalence between HIV and HCV among PWID in rural Puerto Rico does not have a single explanation. Nevertheless, this study found considerable evidence that HCV transmission is occurring in association with the shared use of contaminated cookers, cotton, and water, rather than via syringes. Future research should consider studying HCV infectivity in injection works, like cookers and cottons, following Paintsil et al.’s 2010 study on HCV infectivity in low and high volume syringes [14]. Also, HCV education might not overcome participants’ needs to pool resources. Finally, adding health insurance coverage for HCV treatment, and particularly so for impoverished Puerto Ricans, might help improve HCV screening and testing, diminishing the pool of HCV-infected PWID and, in turn, slowing the spread and lowering the overall prevalence of HCV among this population.

## Additional file

Additional file 1: Table S1. HCV+ result logistic regression (*n* = 308). (DOCX 21 kb)
