# Supplementary material for: Understanding differences in HIV/HCV prevalence according to differentiated risk behaviors in a sample of PWID in rural Puerto Rico
Source: Harm Reduct J. 2016 Mar 8;13:10. doi: 10.1186/s12954-016-0099-9 (PMC4784433; doi:10.1186/s12954-016-0099-9)
Supplement: Additional file 1: Table S1. — HCV+ result logistic regression (n = 308). (DOCX 21 kb) [file 12954_2016_99_MOESM1_ESM.docx]

| Additional file 1: Table S1: HCV+ Result Logistic Regression (n=308) | | | |  |  |  |  |  |  |  |  |  |  |  |  |  |  |  |  |  |  |  |
| --- | --- | --- | --- | --- | --- | --- | --- | --- | --- | --- | --- | --- | --- | --- | --- | --- | --- | --- | --- | --- | --- | --- |
|  | Exp (B) | | Exp (B) | | Exp (B) | | Exp (B) | | Exp (B) | | Exp(B) | | Exp(B) | | Exp(B) | | Exp(B) | | Exp(B) | | Exp(B) | |
| Male | .608 |  | 0.618 |  | .570 |  | .594 |  | .602 |  | .580 |  | .647 |  | .623 |  | .616 |  | .563 |  | .603 |  |
| Age | .959 | * | 0.958 | * | .962 | * | .962 | * | .960 | * | .967 | * | .964 | † | .963 | * | .958 | * | .966 | † | .961 | * |
| Per Capita Income | 1.000 |  | 1.000 |  | 1.000 |  | 1.000 |  | 1.000 |  | 1.000 |  | 1.000 |  | 1.000 |  | 1.000 |  | 1.000 |  | 1.000 |  |
| Married/Living Together | 1.110 |  | 1.092 |  | 1.107 |  | 1.329 |  | 1.125 |  | 1.093 |  | 1.115 |  | 1.109 |  | 1.094 |  | 1.126 |  | 1.291 |  |
| HS Graduate or more | 1.310 |  | 1.289 |  | 1.291 |  | 1.375 |  | 1.306 |  | 1.344 |  | 1.345 |  | 1.376 |  | 1.298 |  | 1.273 |  | 1.379 |  |
| # of Years Injecting Drugs | 1.143 | *** | 1.143 | *** | 1.139 | *** | 1.146 | *** | 1.143 | *** | 1.141 | *** | 1.140 | *** | 1.141 | *** | 1.143 | *** | 1.139 | *** | 1.148 | *** |
| **Past Year:** |  |  |  |  |  |  |  |  |  |  |  |  |  |  |  |  |  |  |  |  |  |  |
| Frequency of Injection | 1.001 |  |  |  |  |  |  |  |  |  |  |  |  |  |  |  |  |  |  |  |  |  |
| # of People Used Needle |  |  | 0.978 |  |  |  |  |  |  |  |  |  |  |  |  |  |  |  |  |  |  |  |
| # of People Used Works |  |  |  |  | 1.029 |  |  |  |  |  |  |  |  |  |  |  |  |  |  |  |  |  |
| # of People Backloaded |  |  |  |  |  |  | 1.114 |  |  |  |  |  |  |  |  |  |  |  |  |  |  |  |
| Freq. of Used Needle Utilization |  |  |  |  |  |  |  |  | 1.094 |  |  |  |  |  |  |  |  |  |  |  |  |  |
| Freq. of Used Cooker Utilization |  |  |  |  |  |  |  |  |  |  | 1.382 | * |  |  |  |  |  |  |  |  |  |  |
| Freq. of Used Cotton Utilization |  |  |  |  |  |  |  |  |  |  |  |  | 1.301 | p=.119 | |  |  |  |  |  |  |  |
| Freq. of Used Water Utilization |  |  |  |  |  |  |  |  |  |  |  |  |  |  | 1.235 |  |  |  |  |  |  |  |
| **Past Month:** |  |  |  |  |  |  |  |  |  |  |  |  |  |  |  |  |  |  |  |  |  |  |
| # of People Used Needle |  |  |  |  |  |  |  |  |  |  |  |  |  |  |  |  | 0.933 |  |  |  |  |  |
| # of People Used Works |  |  |  |  |  |  |  |  |  |  |  |  |  |  |  |  |  |  | 1.176 | * |  |  |
| # of People Backloaded |  |  |  |  |  |  |  |  |  |  |  |  |  |  |  |  |  |  |  |  | 1.143 |  |
| Constant | 2.207 |  | 2.32 |  | 1.942 |  | 1.559 |  | 2.083 |  | 1.211 |  | 1.402 |  | 1.567 |  | 2.357 |  | 1.491 |  | 1.719 |  |
